# multiWGCNA: an R package for deep mining gene co-expression networks in multi-trait expression data

**DOI:** 10.1186/s12859-023-05233-z

**Published:** 2023-03-24

**Authors:** Dario Tommasini, Brent L. Fogel

**Affiliations:** 1 Department of Neurology, UCLA David Geffen School of Medicine, University of California, Los Angeles, 695 Charles E. Young Drive South, Gonda Room 6554A, Los Angeles, CA 90095 USA; 2 Department of Human Genetics, UCLA David Geffen School of Medicine, University of California, Los Angeles, Los Angeles, CA USA; 3 Bioinformatics Interdepartmental Program, University of California, Los Angeles, Los Angeles, CA USA

**Keywords:** Co-expression network, WGCNA, Neurological disease, Preservation, Differential co-expression, Astrocyte reactivity, EAE, rTg4510, Tau pathology

## Abstract

**Background:**

Gene co-expression networks represent modules of genes with shared biological function, and have been widely used to model biological pathways in gene expression data. Co-expression networks associated with a specific trait can be constructed and identified using weighted gene co-expression network analysis (WGCNA), which is especially useful for the study of transcriptional signatures in disease. WGCNA networks are typically constructed using both disease and wildtype samples, so molecular pathways associated with disease are identified. However, it would be advantageous to study such co-expression networks in their disease context across spatiotemporal conditions, but currently there is no comprehensive software implementation for this type of analysis.

**Results:**

Here, we introduce a WGCNA-based procedure, multiWGCNA, that is tailored to datasets with variable spatial or temporal traits. As well as constructing the combined network, multiWGCNA also generates a network for each condition separately, and subsequently maps these modules between and across designs, and performs relevant downstream analyses, including module-trait correlation and module preservation. When applied to astrocyte-specific RNA-sequencing (RNA-seq) data from various brain regions of mice with experimental autoimmune encephalitis, multiWGCNA resolved the de novo formation of the neurotoxic astrocyte transcriptional program exclusively in the disease setting. Using time-course RNA-seq from mice with tau pathology (rTg4510), we demonstrate how multiWGCNA can also be used to study the temporal evolution of pathological modules over the course of disease progression.

**Conclusion:**

The multiWGCNA R package can be applied to expression data with two dimensions, which is especially useful for the study of disease-associated modules across time or space. The source code and functions are freely available at: https://github.com/fogellab/multiWGCNA.

**Supplementary Information:**

The online version contains supplementary material available at 10.1186/s12859-023-05233-z.

## Introduction

Network analysis has been widely applied to gene expression data as a systems genetics approach to understand the coordinated activity of many genes [1]. For this purpose, weighted gene co-expression network analysis (WGCNA) is a popular tool that has been used to identify development-specific transcriptional programs [2], regional [3] and pathological [4] gene networks, and much more.

In a typical WGCNA, to resolve disease-specific alterations, both wildtype and disease samples are used for network construction, using correlation to binary disease status to resolve co-expression networks associated with disease. This approach is particularly effective at identifying cases where disease alters a critical network’s expression level. However, since both wildtype and disease samples are used for network construction, this approach provides no information on changes in network topology, such as whether the network is differentially preserved across conditions [5]. Furthermore, for many disorders, it would be especially useful to analyze how the associated biological networks behave across various conditions beyond simply expression levels. Two key examples of this would be how the module changes across space (e.g., anatomical location, cell type, etc.) or time (e.g., timepoint, age).

When expression profiling experiments include two or more sample traits, multiple experimental designs are possible, and sample selection for network construction becomes more complex. In these cases, most protocols opt for using the entire dataset—regardless of disease status, time, age, tissue, etc.—for network construction [4, 6]. This is reasonable, as correlation-based methods benefit from larger sample sizes. However, other valid designs are possible, such as: (1) subsetting by disease status [4], which enables study of the secondary trait across wildtype and disease samples separately, or (2) subsetting by the secondary trait itself, which enables study of disease across this other trait, which is useful for considerations involving varying space or time. A biologically relevant module should be robust and detectable across these different experimental designs [7]. Currently, a comprehensive software implementation for this type of analysis is not available.

To address this gap, we developed a software tool that constructs all the individual networks for each possible design and subsequently integrates the resulting networks between these designs. Using astrocyte-specific RNA-seq from various brain regions of mice with experimentally induced autoimmune encephalitis (EAE) [8], we demonstrate that our approach can identify disease-specific network topology that is highly relevant to EAE pathology. We also show that this procedure can facilitate selection of biologically interesting modules, a laborious step of standard network analysis. Lastly, we demonstrate how multiWGCNA can be used to study the evolution of pathological modules over time using a previously published module associated with tau accumulation in the rTg4510 mouse model [6].

## Results

### The multiWGCNA workflow

**Fig. 1:**
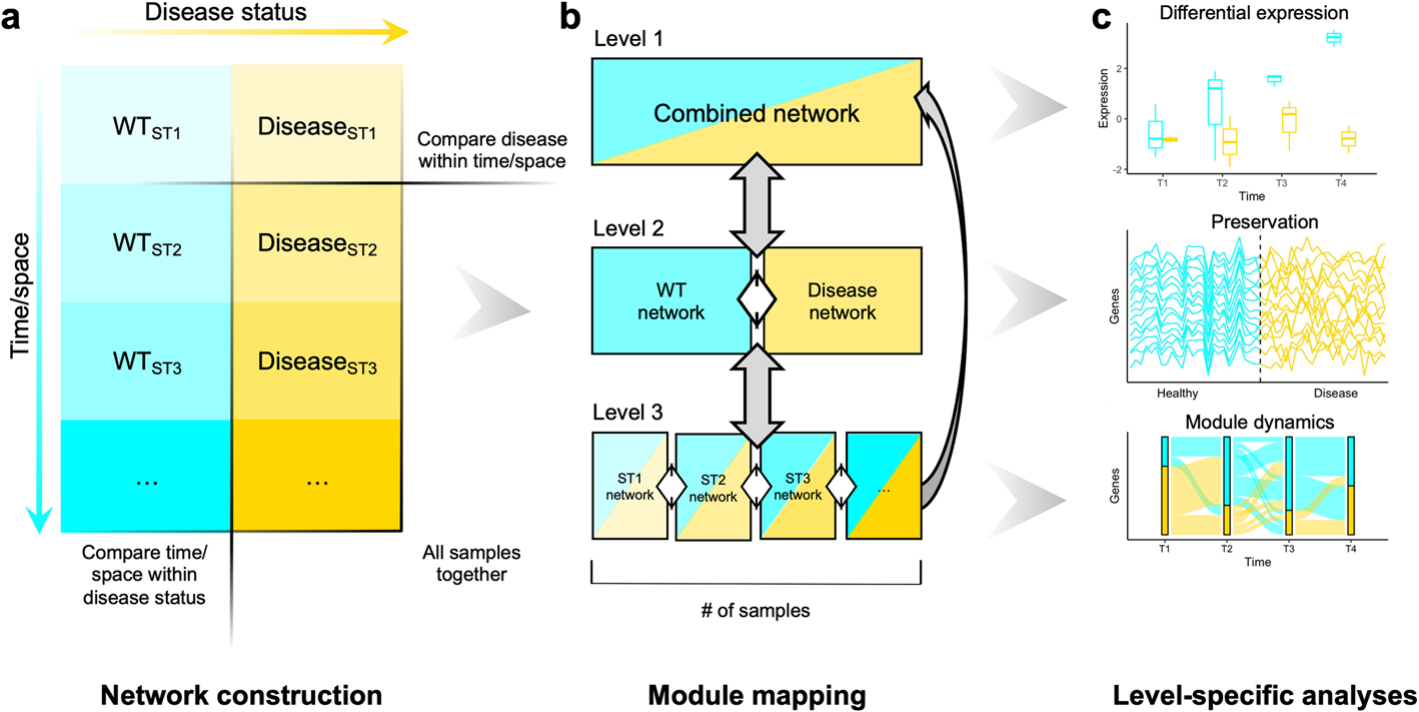
Overview of the multiWGCNA workflow. (**a**) multiWGCNA requires a dataset with two sample traits, such as disease versus a secondary trait like time or space. From this data, co-expression networks are constructed using WGCNA. (**b**) Three levels of networks are constructed from this design: (1) the combined network, (2) the disease and wildtype (WT) networks, and (3) the secondary trait (ST) networks. From these networks, modules can be mapped both across levels (gray arrows) and within levels (white arrows). (**c**) For each network level, appropriate analysis is performed, including differential module expression, module preservation, and module dynamics

We devised a WGCNA-based procedure that can leverage the multidimensionality of experimental designs to study co-expression networks across variable conditions, such as space or time. As shown in Fig. 1a, a typical multidimensional dataset has two sample traits: one of which is often disease status, and the other which is usually space (tissue/region) or time (timepoint/age). We refer to this additional trait as the secondary trait (ST). Broadly, the workflow consists of network construction, module mapping, and level-specific analyses (Fig. 1). In the first step, we perform WGCNA to construct the networks (Fig. 1a), which yields three levels of networks: (1) the combined network, (2) the disease-status networks, and (3) the ST networks. After network construction, we perform module mapping, which maps all modules across and between levels 1, 2, and 3 using module member overlap as a measure of correspondence (Fig. 1b). As shown in Fig. 1b, there are cross-design comparisons (gray) and between-design comparisons (white). Unlike between-design module pairs, cross-design pairs are confounded by shared biological samples and therefore their gene overlap cannot be used to assess biological relationships. However, these comparisons are useful in mapping modules so that a specific network can be traced at each design level. Lastly, we perform level-specific analyses, including module-trait association, module preservation, and analysis of module dynamics (Fig. 1c).

For convenience, module-trait association is performed in level 1 (the combined network), as it includes all samples and conditions. We summarize module expression using the first principal component (module eigengene) and apply the linear model, $Module\,eigengene = disease\,status + secondary\,trait + disease\,status*secondary\,trait$, and significance for each term is tested using factorial ANOVA as has been done previously [6]. However, it is important to note that performing ANOVA using module eigengenes can potentially be misleading as eigengenes are a univariate projection of multivariate data [9]. To address this, we provide users with the option of using PERMANOVA (Permutational Multivariate Analysis of Variance), which provides a non-parametric multivariate analysis of variance [10], as a means for performing multivariate data comparisons in complex experimental designs. Additional multivariate analytic methods, such as the R package resampleWGCNA [9], could also be employed at the user’s discretion.

In level 2 and 3 networks, we study module preservation and module dynamics. Preservation analysis is performed within each level—for example, using the disease networks as reference and the wildtype data as test or vice versa. Based on the previous implementation [5], we define a differentially preserved module as any module with low or no preservation (Z_summary_ < 10) in the test dataset. To test if preservation scores are lower than expected by chance, we implemented a permutation test (see Methods) that can be used to test the probability of observing the biological preservation score when phenotype labels are randomly assigned. Module dynamics is a more subtle analysis that consists of analyzing the flow of module genes across conditions to identify interesting module-membership patterns.

### multiWGCNA identifies an EAE-induced network in astrocytes

**Fig. 2:**
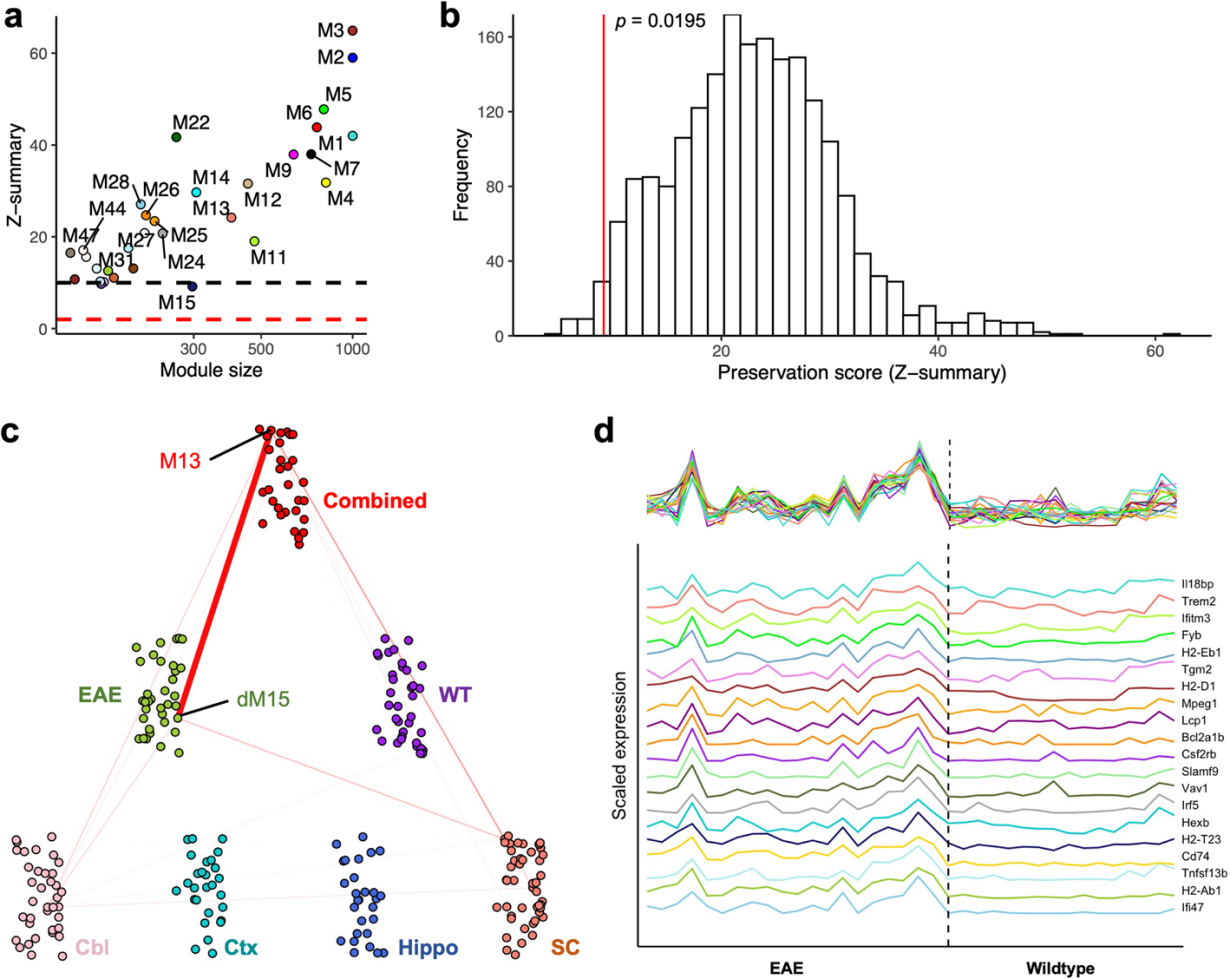
multiWGCNA reveals an EAE-specific network in astrocytes. (**a**) Preservation scores of modules from EAE network in wildtype network, using astrocyte Ribotag data. (**b**) Distribution of expected preservation scores for modules of similar size to dM15 when phenotype labels are randomly assigned (2000 permutations). (**c**) Network view of the correspondence (based on hypergeometric overlap *p* value) between dM15 and the other modules constructed by multiWGCNA. (**d**) Expression patterns of the top 20 connected genes in dM15, showing highly unsynchronized expression in the wildtype samples

To test whether multiWGCNA can reveal biologically meaningful relationships from expression profiling experiments, we performed multiWGCNA on an astrocyte-specific Ribotag dataset that compared mice with EAE (n = 20) to wildtype controls (n = 16) across the cerebral cortex, cerebellum, hippocampus, and spinal cord [8]. After filtering modules driven by a single sample (see Methods), multiWGCNA identified a single module from the disease network (dM15, d = disease network) that exhibited weak preservation (Z_summary_ = 9.16) in wildtype astrocytes (Fig. 2a). Permutation testing with random phenotype assignment revealed that the preservation score of dM15 is significantly lower than that expected by chance (*p* = 0.0195 by 2000 permutations, Fig. 2b). Using a subsampling procedure, we independently validated that the sample size of the wildtype dataset (n = 16) was large enough to obtain accurate preservation scores (see Additional file 1: Fig. S1, see Methods).

In addition, dM15 could only be detected in the combined network (M13, FDR = 3.8 × 10^–306^, 249/303 genes), but not the wildtype network, or any region-specific network (Fig. 2c), and thus appears to require many EAE astrocyte samples for detection. Importantly, genes in dM15 show tightly coordinated expression in EAE astrocytes, but largely unsynchronized expression in wildtype astrocytes (Fig. 2d). In agreement with these results, we performed differential co-expression analysis [11] and found that 99.86% (5894/5902) differentially co-expressed pairs (FDR < 0.05) of module genes had higher co-expression in EAE astrocytes than wildtype astrocytes (see Additional file 1: Fig. S2A). In contrast, no differentially co-expressed genes between disease and wildtype conditions were observed for a similarly sized module, dM14 (see Additional file 1: Fig. S2B). Therefore, multiWGCNA identified a co-expression network that is induced in astrocytes by EAE.

### multiWGCNA improves prioritization of key biological networks

**Fig. 3:**
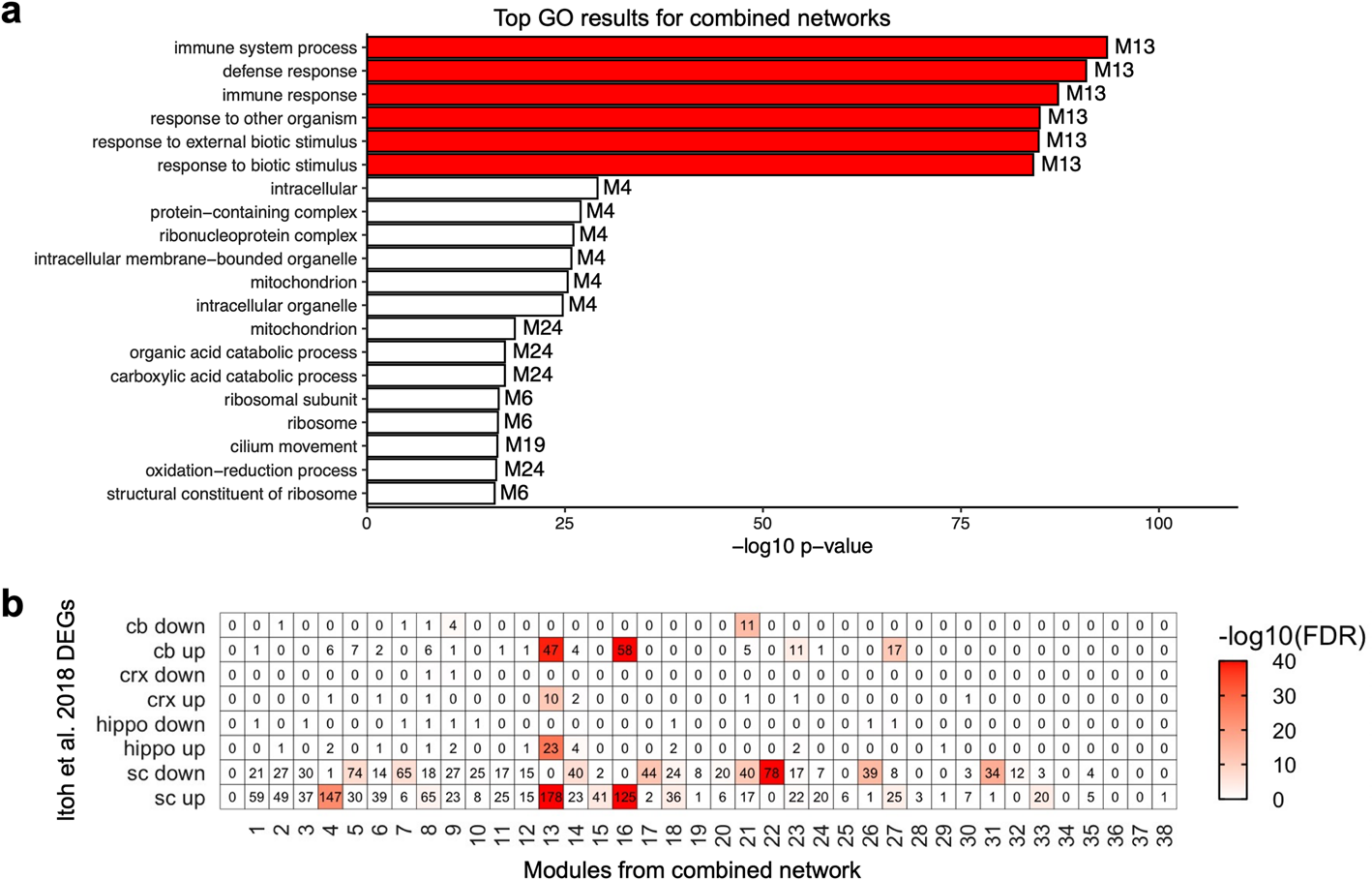
The module identified by multiWGCNA is the most biologically relevant to EAE. (**a**) Top GO terms for all modules of the combined EAE network. A maximum of 6 terms were allowed per module. M13/dM15 dominates the top of the list and has terms most relevant to EAE. (**b**) Overlap analysis between combined EAE network modules and DEGs from Itoh et al. [8]. Only M13 is enriched for genes upregulated in EAE across all CNS regions profiled

Selection and prioritization of co-expression networks remains a laborious step of the standard workflow. If one were to use only the combined network as in standard WGCNA, there are two modules (M13 and M16) that have a significant association to EAE or the interaction between EAE and region at an FDR < 0.05, and six modules (M13, M16, M22, M33, M32, M20) that have a significant association to EAE at an FDR < 0.1 by factorial ANOVA (see Additional file 1: Table S1). However, multiWGCNA clearly indicates that M13 in the combined network (dM15 in the disease network) is the module most relevant to EAE, as it is the only module with a disease-specific co-expression pattern (Fig. 2a). Since modules are thought to represent shared biological processes, we used gene ontology (GO) [12] to validate that the module identified using multiWGCNA is indeed the most relevant EAE-associated network. Indeed, M13 dominated the top of the enrichment results and was enriched for GO terms like immune system process and defense response, which are highly relevant to EAE pathology (Fig. 3a). We also cross-referenced our modules with the original published lists of differentially expressed genes (DEGs) [8]. Strikingly, M13 was the co-expression network that best defined the EAE-induced transcriptional response of astrocytes across all four regions profiled, suggesting that the transcriptional responses of astrocytes to EAE across the CNS are actually part of a convergent molecular pathway that can be delineated by a single co-expression network (Fig. 3b). Ultimately, these findings indicate that multiWGCNA can indeed facilitate the module selection step of the network analysis workflow, rapidly identifying biologically-relevant disease-associated networks across anatomical regions.

### The module identified by multiWGCNA corresponds to the A1 neurotoxic phenotype

**Fig. 4:**
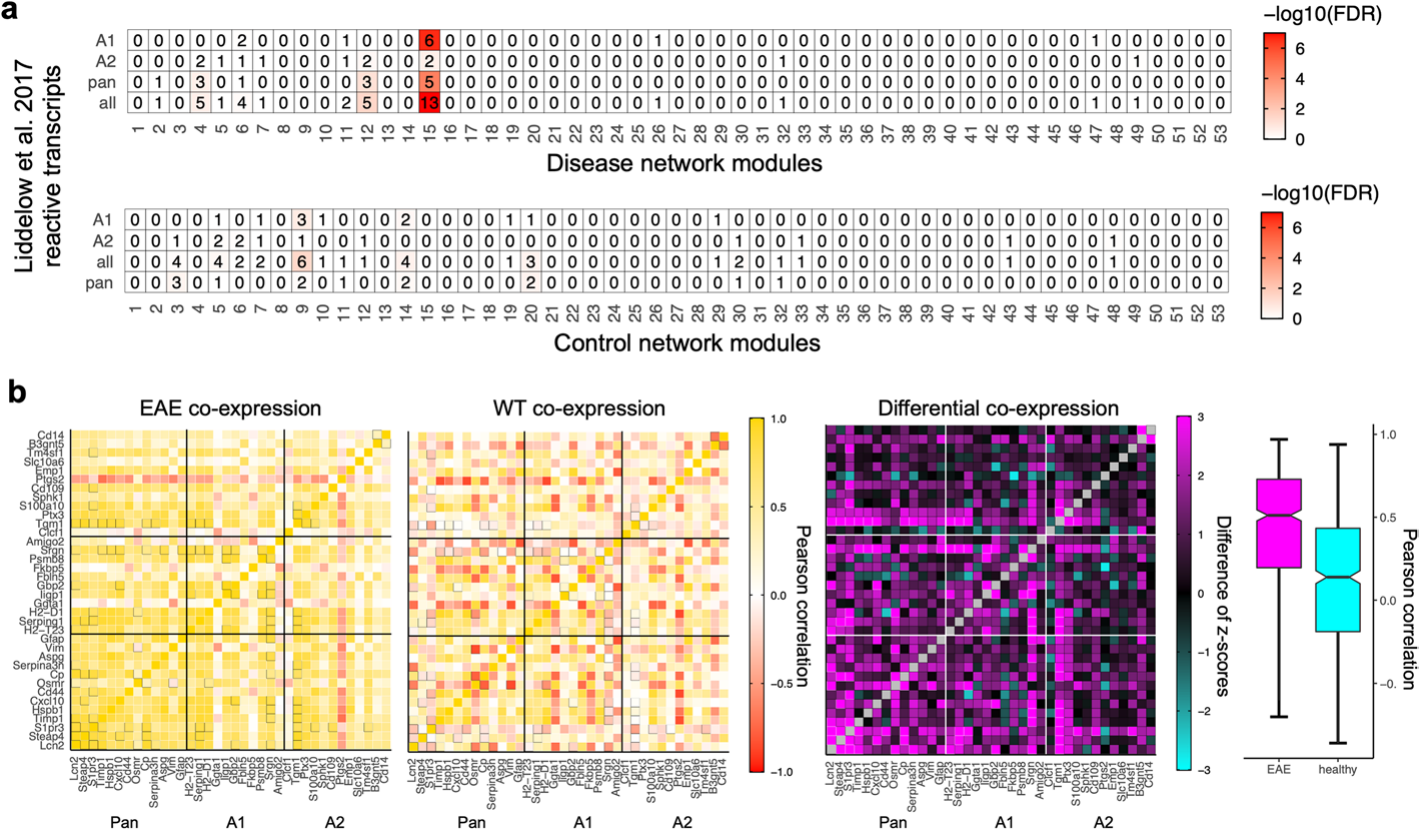
The immune astrocyte module corresponds to the A1 neurotoxic phenotype. (**a**) Overlap analysis between modules from EAE/wildtype networks and the reactive astrocyte transcripts used in Liddelow et al. [13]. The only network enriched for reactive astrocyte transcripts across all disease and wildtype modules is dM15. (**b**) Left heatmap shows the Pearson correlation between reactive astrocyte transcripts in the EAE astrocyte samples from Itoh et al. [8]. Middle heatmap shows the Pearson correlation between reactive astrocyte transcripts in the wildtype astrocyte samples from Itoh et al. [8]. Right heatmap shows the differential co-expression of reactive astrocyte transcripts between EAE and wildtype conditions, where high z-scores represent higher co-expression in EAE. Boxes around heatmap cells signify significantly different correlations (FDR < 0.05). Right boxplots show the quantification of the Pearson correlations from the first two heatmaps

Since multiple sclerotic lesions are specifically associated with astrocytes that have undergone reactive astrocytosis [8, 13], we hypothesized that M13/dM15 might correspond to the reactive astrocytic cell state. We used the reactive astrocyte transcriptional markers from Liddelow et al. [13], which included transcripts specific to neurotoxic A1 and neuroprotective A2 astrocytes as well as pan-reactive transcripts. We confirmed these reactive astrocyte transcripts exhibited higher expression in EAE astrocytes, suggesting that gliosis can be detected in the Ribotag dataset (see Additional file 1: Fig. S3). Next, we tested whether our EAE modules were enriched for reactive astrocyte markers (Fig. 4a). dM15 was highly enriched for all 36 reactive astrocyte markers (FDR = 1.0 × 10^–13^, 13/36 genes), and was also enriched for the neurotoxic A1-specific transcripts (FDR = 2.3 × 10^–7^, 6/11 genes) as well as pan-reactive transcripts (FDR = 3.2 × 10^–5^, 5/13 genes). Importantly, no other modules in the EAE network were enriched for any of these gene sets at an FDR of 1%, indicating that dM15 is the network most representative of the reactive astrocyte cell state in the EAE Ribotag data (Fig. 4a). Likewise, no modules from the wildtype network were enriched for reactive astrocyte transcripts, indicating that the reactive astrocyte co-expression signature can only be detected in astrocytes from mice with EAE (Fig. 4a).

Since dM15 was lowly preserved in wildtype astrocytes, we reasoned that the reactive astrocyte-specific transcripts themselves may be differentially co-expressed between wildtype and EAE astrocytes. We performed differential co-expression analysis [11], which found that 53 out of 53 differentially co-expressed pairs of these 36 reactive astrocyte transcripts have higher co-expression (FDR < 0.05) in EAE than in wildtype astrocytes (Fig. 4b). This is especially true of pan- and A1-reactive transcripts, which exhibited a very strong differential co-expression signature, and less so for A2 markers (Fig. 4b). This is in line with the finding that dM15 is enriched for A1- and pan-reactive transcripts but not A2 reactive transcripts (Fig. 4a). In sum, by splitting samples by disease status and performing differential network analysis, multiWGCNA was able to resolve an EAE-induced transcriptional program that corresponds to the neurotoxic A1 phenotype.

### Module dynamics reveals evolution of pathological modules over disease course

**Fig. 5:**
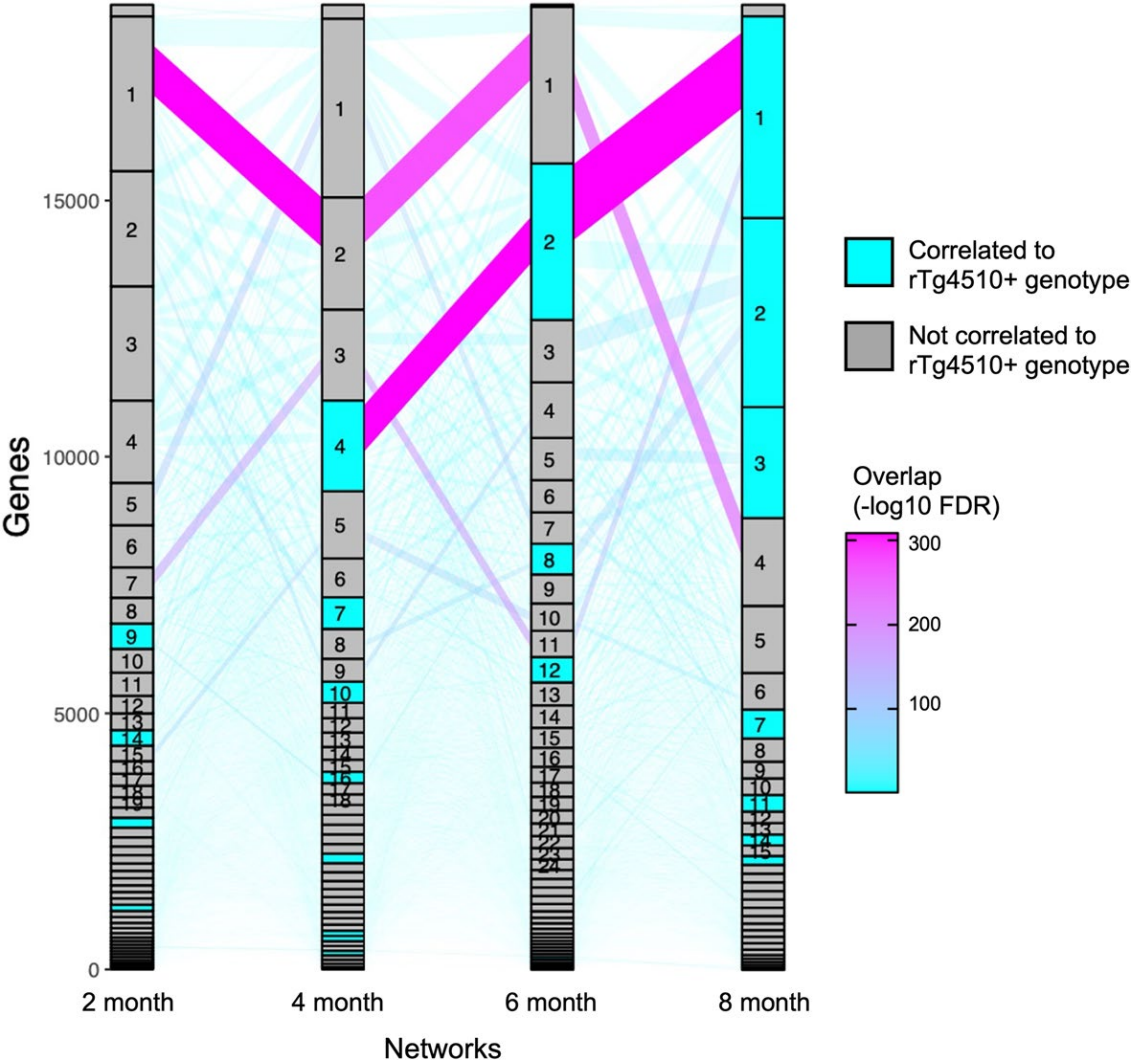
Module dynamics of the timepoint-specific networks from rTg4510 + mice exhibiting tau pathology. Sankey flow diagram showing the transfer of genes from one timepoint to the next timepoint across the 2–8 month timecourse. The y-axis represents genes while the x-axis represents the different timepoint networks in order of time. The color of the edges represents module correspondence (based on hypergeometric overlap *p* value)

Next, we applied multiWGCNA to an RNA-seq dataset from the entorhinal cortex of the rTg4510 mouse model of the tau pathology in Alzheimer’s Disease, with ages ranging from 2 to 8 months, covering the onset of tau accumulation in the cortex [6]. In the original study, a WGCNA module turquoise was identified as being positively correlated to tau accumulation in both the entorhinal cortex and the hippocampus as measured by immunohistochemistry [6]. Since we used the same parameters as the original paper (see Methods), the modules from our combined network (n = 58) were identical to theirs (see Additional file 2). To resolve temporal network changes related to tau pathology, we focused on the level three networks, in which each timepoint (2, 4, 6, and 8 month) is subjected to an individual network analysis (n = 15, 15, 13, and 15, respectively). Overlap analysis revealed that the turquoise module (M1 in the combined network) is the equivalent of module 4m-4 in the 4 month network (FDR = 3.4 × 10^–306^, 1110/3091 genes), 6m-2 in the 6 month network (FDR = 6.1 × 10^–306^, 1680/3091 genes), and 8m-1 (FDR = 4.5 × 10^–306^, 1825/3091 genes) in the 8 month network, but has no clear corresponding module in the 2 month network. Comparative analysis of these level three networks (Fig. 5) revealed that 4m-4, 6m-2, and 8m-1 correlate with disease. Interestingly, these late timepoint modules have no clear origin in the 2 month network.

**Fig. 6:**
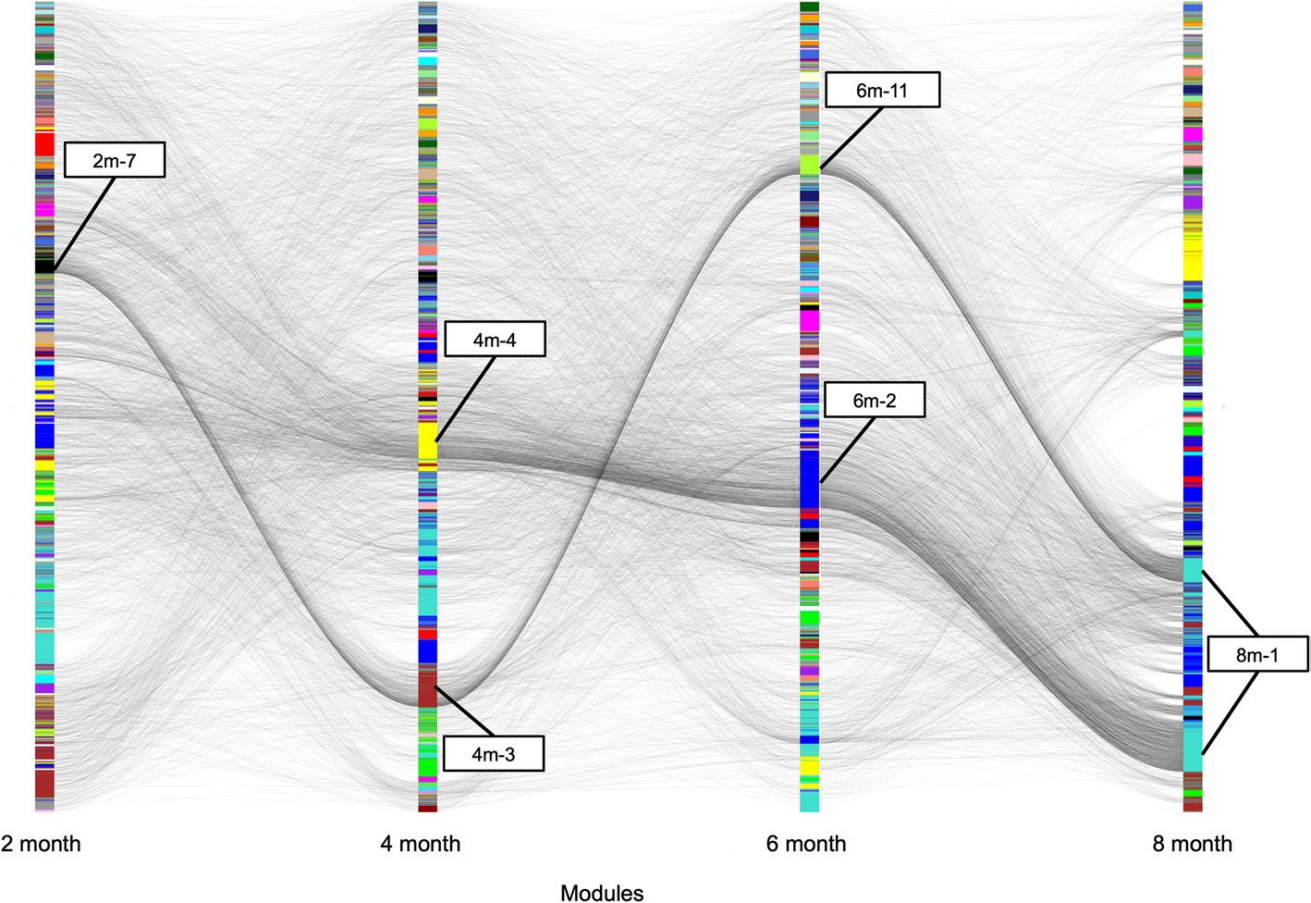
Genesis of the module associated with tau pathology mimics tau burden in the entorhinal cortex. Gene-level sankey flow plot showing flow of genes originating from 8 month equivalent of the turquoise module (8m-1) from Castanho et al. [6]. The topological clustering tree for each timepoint network is shown along with module colors. Most of the gene recruitment to the turquoise module occurs at 4 months of age, which is the onset of tau pathology in the entorhinal cortex

To get gene-level resolution, we plotted the topological overlap clustering trees for the four networks constructed with wildtype and rTg4510 mice at 2, 4, 6, and 8 months (Fig. 6). Figure 6 shows where the genes of the 8 month version of the turquoise module (8m-1) are located in the gene clustering of earlier timepoints. This revealed a stepwise recruitment of genes to this pathological cluster over the course of tau pathology, such that peripheral genes from other modules are recruited to become part of the turquoise network (Fig. 6). The hubs are well-preserved from 4 to 8 months (see Additional file 1: Fig. S4-6, also see Additional file 3). At 2 months of age, turquoise genes are interspersed across the topological overlap clustering tree, but their position in the tree is greatly refined at 4 months of age, indicating that the formation of the turquoise module follows tau accumulation, which is also detected in the entorhinal cortex at 4 months of age [6]. Another interesting feature revealed by the analysis in Fig. 6 is that part of the 8m-1 module originates from a group of genes that is tightly connected throughout the time-course. These genes are enriched for GO terms blood vessel development (*p* = 1.28 × 10^–13^) and cell migration (*p* = 5.30 × 10^–13^), and only join this pathological module at month 8 (Fig. 6). Interestingly, the late timepoint modules are all well-preserved at 2 months (8m-1 Z_summary_ = 26.5; 6m-2 Z_summary_ = 28.3; 4m-4 Z_summary_ = 25.5) suggesting that the turquoise module is a constitutive transcriptional network that may be rewired in disease rather than developing de novo like M13/dM15 in EAE. Ultimately, these are subtleties of module dynamics that would be entirely missed when performing a standard WGCNA.

## Discussion

In summary, our proposed software tool, multiWGCNA, performs all the network constructions possible in a multi-trait expression dataset to maximize the amount of data extracted from large-scale RNA-seq experiments and identify interesting co-expression relationships. This package provides several key features that build on the existing WGCNA R package [1]. A notable advantage of multiWGCNA is that it uses a linear model and ANOVA to identify significant associations between module expression and disease status along with a secondary spatiotemporal trait, functions which are not directly available in the standard WGCNA package. The multiWGCNA tool also provides a comprehensive, automated workflow for resolving modules that have trait-specific expression, preservation, and gene recruitment (described in Fig. 1), and can be easily integrated into existing WGCNA pipelines without a need for detailed customization. Lastly, multiWGCNA provides additional useful functions for data visualization. These include module tracing diagrams (Fig. 2c, available through the function drawMultiWGCNAnetwork), which constructs a graph of all modules clustered by design level where edges signify correspondence between a module of interest and modules from other designs. multiWGCNA also provides module dynamics plots (Fig. 5, available through the function moduleComparisonPlot), which draw a sankey diagram of the movement of genes across networks. Lastly, the TOM-flow plot (Fig. 6, available through the function TOMFlowPlot) allows users to track the movement of individual genes across the topological overlap clustering tree of different networks. Collectively, these functions complement the purpose of the WGCNA package by extending the analysis and visualization of co-expression networks across multiple dimensions in a comprehensive, accessible, and easily integrated format for both new and sophisticated WGCNA users.

When considering the methodology behind the development of the networks, given that we find WGCNA to be very sensitive to the state of the biological samples used for network construction, other network-based methods may also generate different results depending on the tissue, timepoint, and/or treatment groups included in the analysis. Therefore, the subset-and-integrate procedure we apply here might prove useful for such methods as well. Furthermore, such integration could prove to be complimentary to the analysis. For example, since WGCNA does not aim to identify causal regulatory relationships, the network construction procedure we describe could instead be applied using one of the various gene regulatory network inference algorithms currently available [14–17] for network construction instead of WGCNA, potentially revealing regulatory interactions that may be conditional on a specific sample trait. Although such integration is not presently incorporated in the multiWGCNA package, it could be added to future implementations.

We provide two examples of how multiWGCNA can be applied to real experimental data. Importantly, we show that multiWGCNA is able to resolve the induction of reactive astrocytes in EAE, a well-established feature of EAE pathology. We note that, while the network identified by multiWGCNA is highly similar to that identified by the standard workflow, there are two important differences. First of all, we show that selection of interesting modules is more rapidly achieved through multiWGCNA (Figs. 2, 3). Second and more importantly, the interpretation of the multiWGCNA results is quite different from the interpretation of the results from the combined network alone. In standard WGCNA, the module derived from the combined network (M13) is presumed to be preserved in the entire dataset as both wildtype and EAE samples were used for network construction. However, in multiWGCNA, we construct separate networks for EAE and wildtype samples, which reveals that M13 is recapitulated only in EAE astrocytes (dM15) and not in wildtype astrocytes (Fig. 2c). Together with the finding that dM15 is differentially preserved, our results indicate that M13/dM15 is a de novo disease-associated network that is gained in EAE. multiWGCNA can therefore differentiate biological pathways as being over- or underexpressed versus being entirely inactive under different experimental conditions related to disease. It should be noted that in bulk RNA-sequencing from heterogenous tissue, modules are often interpreted as reflecting different cell types [3]. Thus, it is rather appropriate that in an astrocyte-specific Ribotag dataset, the pathological module identified maps to a specific cell subtype—neurotoxic A1 astrocytes—rather than a cell type, as the expression data itself is already astrocyte-specific.

Interestingly, the authors of the original EAE Ribotag dataset identified cholesterol biosynthesis as a therapeutic target in EAE and were able to mitigate symptoms by increasing efflux of cholesterol to extracellular apolipoprotein (APOE) [8]. It was recently discovered that neurotoxic reactive astrocytes secrete saturated lipids in APOE and APOJ lipoparticles that induce cell death in neurons and oligodendrocytes [18]. Thus, it may be that the biosynthesis pathway in A1 neurotoxic astrocytes is redirected to produce these toxic lipids at the expense of normal cholesterol biosynthesis, explaining the downregulation of this pathway. This would suggest that A1 astrocyte reactivity has a two-pronged deleterious effect: (1) depletion of the cholesterol that neurons and oligodendrocytes rely on for building synapses and myelin respectively [8], and (2) secretion of saturated lipids that directly kill neurons and oligodendrocytes [18]. These observations may also explain why eliminating long-chain saturated lipids only partially reduces astrocyte-mediated cell death after axon crush, while preventing neurotoxic astrocyte induction by genomic deletion of *Il1a*, *Tnf* and *C1qa* completely reduced astrocyte-mediated cell death [18].

Naturally, the question arises of which network constructed by multiWGCNA is the most accurate. In our opinion, it depends on the biological question at hand. In the case of M13/dM15, the level 2 EAE network (dM15) is more biologically interesting as its co-expression structure is not obfuscated by the wildtype samples. In cases where it is less obvious, we suggest use of the key module(s) derived from the combined network for a larger sample size and more robust correlations. Ultimately, this may be of less importance because the modules should be highly similar and downstream analyses will yield almost identical results. In general, we recommend large sample sizes to allow enough power (at least 12 samples per condition) for level 2 and level 3 analyses.

In this report, we also introduce the idea of module dynamics, and demonstrate its use on time-course data in a module correlated with tau accumulation in the rTg4510 mouse model of tau pathology (Fig. 5). The pathological transformation of this module, both in terms of disease correlation (Fig. 5) and gene recruitment (Fig. 6), is mirrored by tau accumulation in the entorhinal cortex and hippocampus, which also occurs at 4 months of age.

In summary, we have developed a WGCNA software tool, multiWGCNA, that is tailored to analyzing multi-trait expression data. Because this approach is especially powerful at analyzing biological networks with temporal or spatial variance, we recommend multiWGCNA for future studies that include these conditions in their experimental design.

## Conclusion

The multiWGCNA R package provides a comprehensive set of high-level software tools for analyzing co-expression networks in datasets where samples have two variable traits. We expect the multiWGCNA R package will reveal interesting and novel network characteristics associated with spatical or temporal aspects, such as anatomy, development, and/or pathology.

## Materials and methods

### Module nomenclature

For combined networks, we named each modules using a capital M followed by the numeric identifier from the combined network (i.e. M13). For disease networks, we added c- to signify control and a d- prefix to signify disease (i.e. dM15). Level three networks use a prefix (i.e. 8m-1) to signify the originating WGCNA dataset.

### Permutation testing to determine trait-specific preservation

The standard WGCNA framework [5] determines the degree to which a module is preserved by computing a Z_summary_ score that combines several measures of preservation. In the case of multiWGCNA, to confirm biological relevance, we want to determine if a module detected in a subset of the data has lower preservation than expected by chance in a different subset of the data. To this end, we developed a novel permutation test (available using the function diseasePreservationPtest) based on the previous implementation. First, phenotype labels (e.g., disease and wildtype) are randomly assigned to samples, then WGCNA is performed in the randomized reference set (e.g., disease), and preservation of the resulting modules is assessed in the randomized test set (e.g., wildtype) by performing module preservation analysis and retrieving Z_summary_ values. Since Z_summary_ preservation scores tend to be higher for larger modules [5], the null distribution of preservation scores must be calculated using modules of comparable sizes. Therefore, in each permuted set, we first remove modules driven by single samples (see Materials and Methods) as these outlier modules reflect noise, and we then select the preservation score of the module whose size is closest to the module of interest. This allows us to compute an empirical distribution of preservation scores under the null hypothesis where phenotype has no effect on preservation. A *p* value is computed by taking the proportion of preservation scores that are lower than or equal to the observed score (e.g., one-tailed), as this reflects biological relevance under these conditions.

To illustrate, we applied this permutation procedure to the astrocyte Ribotag data, where we sought to determine if the dM15 module from the EAE network had lower preservation in the wildtype network than expected by chance (see Results). To answer this question, we performed 2000 permutations of the procedure described above. For each permutation, we constructed the reference network from twenty randomly selected samples, tested for network preservation in the remaining sixteen samples, filtered out modules driven by single samples, and extracted the preservation score of the module whose size was closest to dM15 (303 genes) in that network (the resulting modules ranged from 225 to 366 genes in size). This generated a null distribution of preservation scores assuming that EAE status has no effect on network preservation. Out of 2000 permutations, there were 39 scores lower than or equal to the observed score for dM15 (9.16), which corresponds to a *p* value of 0.0195 (Fig. 2b) thus indicating biological significance.

### Analysis of reactive astrocyte markers

36 of the 37 reactive astrocyte markers from Liddelow et al. [13] were used here because Ugt1a1 was not expressed in any sample from the astrocyte Ribotag dataset. Differential co-expression analysis was performed using dcanR [11], which applies Fisher’s transformation to transform correlation coefficients to z-scores and tests for a significant difference in z-scores using the z-distribution. For consistency with the WGCNA networks, we used Pearson correlation as a measure of co-expression for this analysis as well.

### Estimating the minimum number of biological samples required for reliable module preservation analysis

We independently validated that the number of biological replicates used for module preservation analysis in this paper are sufficient to obtain reliable preservation scores of network preservation (see Additional file 1: Fig. S1). Using a large dataset of 100 wildtype mouse hippocampus samples [19, 20], we randomly selected 50 samples to build a reference network. We tested for network preservation in the other half of the dataset (n = 48, two sample outliers were removed). After excluding modules driven by single samples, all large modules (> 150 genes) were strongly preserved (Z_summary_ > 10) and all small modules (< 150 genes) were fairly preserved (Z_summary_ > 8). This is as expected as the test samples were selected from the sample phenotypic group used to build the reference network, so all modules should be preserved. We defined these preservation statistics as the ground truth and asked how small we could make the sample groups until the resulting conclusions deviated from these classifications. We developed a scoring scheme, where modules with Z_summary_ < 2 receive a 0, modules with Z_summary_ > 2 but Z_summary_ < 10 receive a 1, and modules with Z_summary_ > 10 receive a 2 and accuracy is measured by the percent difference between the module preservation score of the subsample relative to the ground truth. This scoring scheme takes into account the degree to which conclusions of preservation differ. We randomly sampled 100 combinations of 24 samples from the 48 test samples and repeated network preservation (see Additional file 1: Fig. S1). We repeated this procedure with sample sizes of 15, 12, 10, 8, and 5 and found that 12 biological samples was the lowest number of samples that could be used while retaining a high classification accuracy (90%).

### multiWGCNA of astrocyte-specific Ribotag data from EAE mouse model

Astrocyte-specific RNA-seq from various regions of the mouse CNS was retrieved from GSE100329. Raw counts were converted to RPKM and log2 transformed. MultiWGCNA was performed using a soft threshold power of 12, a minimum module size of 100 genes, and merge cut height of 0 (no merging) for all seven networks (the combined network, the disease status networks, and the region-specific networks).

### multiWGCNA of entorhinal cortex timecourse data from rTg4510 mouse model

RNA-seq from entorhinal cortex of mice with tau pathology (rTg4510) at various ages was retrieved from GSE125957. The data was processed and WGCNA was performed using the same function and parameters as Castanho et al. [6] described (https://git.exeter.ac.uk/ic322/ad-mice-rna-seq-cell-reports). Briefly, genes with a count sum of less than 7 were removed and the sample outlier, S19, was dropped. For network construction, the blockwiseModules function was run with a soft threshold power of 10, a minimum module size of 30 genes, a max block size of 25,000, and a merge cut height of 0.25. The resulting combined network yielded modules identical to those reported by Castanho et al. [6].

### Filtering modules in multiWGCNA

Modules that are driven by a single sample were filtered out as outlier modules in the multiWGCNA workflow. We define an outlier module as any module where the variance of module eigengenes is less than 0.02 upon removing the sample with the largest absolute module eigengene. We find that this method performs better than interquartile range or z-score based methods for identifying outlier modules.

### Availability and requirements

- Project name: multiWGCNA
- Project home page: https://github.com/fogellab/multiWGCNA
- Operating system(s): Platform independent
- Programming language: R
- Other requirements: none
- License: GNU GPL 3
- Any restrictions to use by non-academics: none

## Supplementary Information

**Additional file 1. **Supplemental Data. Figures S1–S6, Table S1.

**Additional file 2. **Network Comparison. Comparison of multiWGCNA combined network modules to Castanho et al. 2020 standard WGCNA modules.

**Additional file 3. **Module Dynamics. MultiWGCNA temporal gene rank comparisons related to Castanho et al. 2020 data.

## Data Availability

The datasets used and/or analyzed during the current study are available from the corresponding author on reasonable request.

## Abbreviations

EAE: Experimental autoimmune encephalitis
GO: Gene ontology
RNA-seq: RNA-sequencing
ST: Secondary trait
WGCNA: Weighted gene co-expression network analysis
