# Supplementary material for: multiWGCNA: an R package for deep mining gene co-expression networks in multi-trait expression data
Source: BMC Bioinformatics. 2023 Mar 24;24:115. doi: 10.1186/s12859-023-05233-z (PMC10039544; doi:10.1186/s12859-023-05233-z)
Supplement: Supplementary file 1 — Additional file 1. Supplemental Data. Figures S1–S6, Table S1. [file 12859_2023_5233_MOESM1_ESM.docx]

**Supplemental Material for multiWGCNA: an R package for deep mining gene co-expression networks in multi-trait expression data**

Dario Tommasini^1^, Brent L. Fogel^1,2,3,4*^

^1^ Department of Neurology, UCLA David Geffen School of Medicine, University of California, Los Angeles

^2^ Department of Human Genetics, UCLA David Geffen School of Medicine, University of California, Los Angeles

^3^ Bioinformatics Interdepartmental Program, University of California, Los Angeles

^*^ corresponding author

**Figure S1. Minimum number of samples required to obtain accurate preservation scores**. A large (n=100) dataset of hippocampus RNA-seq samples was divided in half. The first half was used to construct networks using WGCNA. The entire second half was then used to calculate preservation scores, defined as the ground truth. Then, subsamples of the second half of the dataset were taken and used to calculate preservation. This was performed for sample sizes of 24, 15, 12, 10, 8, and 5. The sample size of 12 was the lowest sample size that still retained a high classification accuracy (>90%).


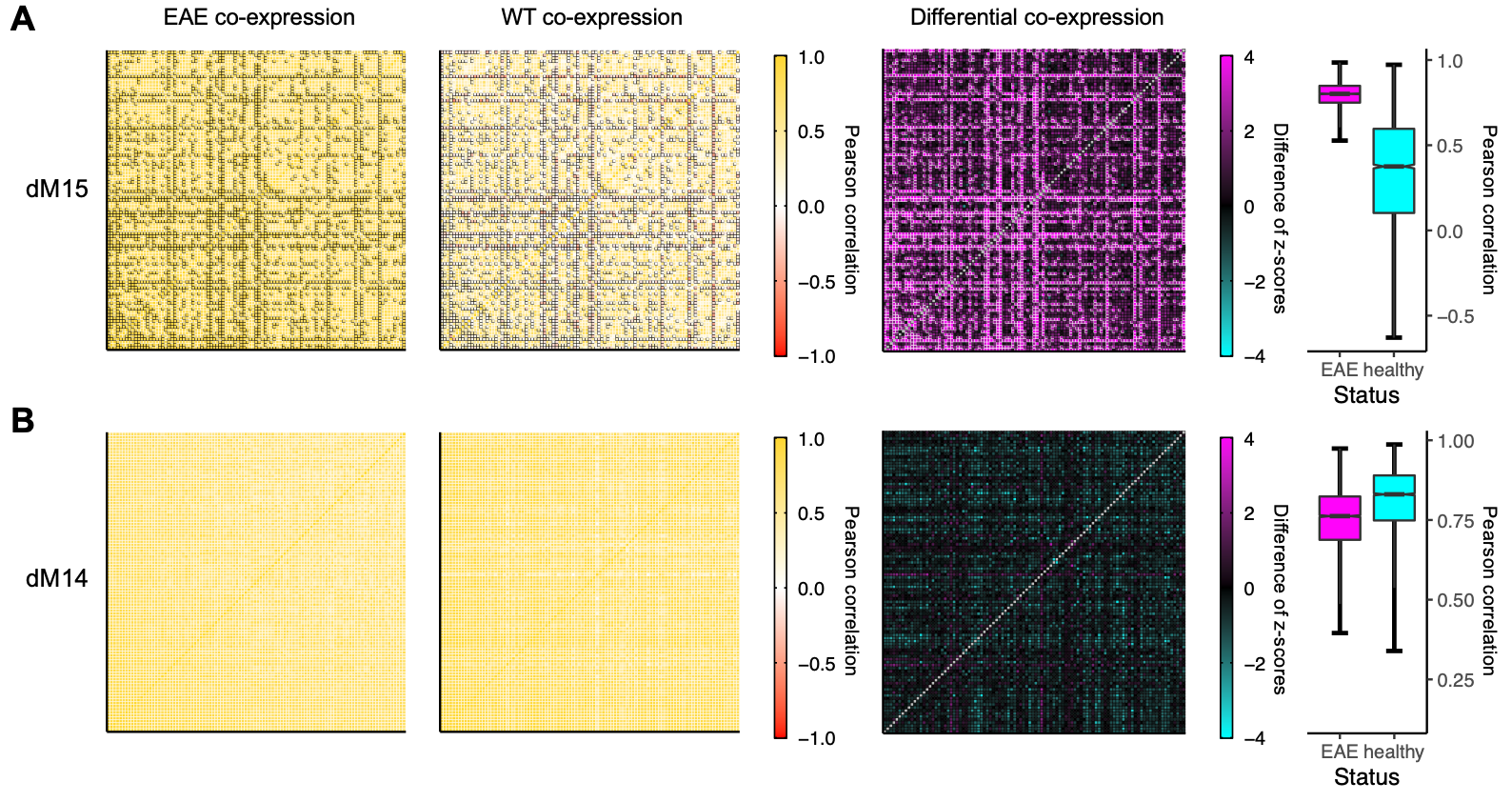


**Figure S2. Genes of dM15 are differentially co-expressed between EAE and wildtype astrocytes**. A) Left heatmap shows the Pearson correlation between the top 100 connected genes of dM15 in the EAE astrocyte samples from Itoh et al. 2018. Middle heatmap shows the Pearson correlation between the top 100 connected genes of dM15 in the wildtype astrocyte samples from Itoh et al. 2018. Right heatmap shows the differential co-expression of the top 100 connected genes of dM15 between EAE and wildtype conditions, where high z-scores represent higher co-expression in EAE. Boxes around heatmap cells signify significantly different correlations (FDR < 0.05). Right boxplots show the quantification of the Pearson correlations from the first two heatmaps. B) Like A but for the top 100 connected genes of dM14, a module of similar size to dM15 that is not differentially co-expressed.

|  | **Disease FDR** | **Disease*Region FDR** | **Region FDR** |
| --- | --- | --- | --- |
| combined_13 | **0.00447553** | 0.98775812 | 0.00057074 |
| combined_16 | **0.05547402** | **0.01966684** | 2.67E-09 |
| combined_22 | **0.05547402** | 0.98775812 | 0.15780287 |
| combined_33 | **0.05547402** | 0.98775812 | 6.35E-06 |
| combined_32 | **0.07597411** | 0.98775812 | 0.12281016 |
| combined_20 | **0.08191645** | 0.98775812 | 1.63E-05 |
| combined_26 | 0.12554887 | 0.98775812 | 0.0227402 |
| combined_05 | 0.12929057 | 0.98775812 | 1.28E-18 |
| combined_17 | 0.12929057 | 0.98775812 | 0.00339049 |
| combined_31 | 0.12929057 | 0.98775812 | 8.94E-06 |
| combined_03 | 0.19947748 | 0.98775812 | 5.14E-14 |
| combined_04 | 0.19947748 | 0.98775812 | 0.00823777 |
| combined_02 | 0.20151948 | 0.98775812 | 7.96E-13 |
| combined_09 | 0.20151948 | 0.98775812 | 1.84E-24 |
| combined_21 | 0.21156074 | 0.98775812 | 1.81E-17 |
| combined_15 | 0.27472467 | 0.98775812 | 0.00128614 |
| combined_10 | 0.28574653 | 0.98775812 | 1.72E-05 |
| combined_11 | 0.28680802 | 0.98775812 | 2.88E-19 |
| combined_07 | 0.29811413 | 0.98775812 | 9.97E-05 |
| combined_14 | 0.37794701 | 0.98775812 | 7.31E-22 |
| combined_24 | 0.37794701 | 0.98775812 | 3.63E-09 |
| combined_12 | 0.38637708 | 0.98775812 | 4.94E-17 |
| combined_25 | 0.38637708 | 0.98775812 | 0.17862479 |
| combined_01 | 0.40639801 | 0.98775812 | 4.94E-17 |
| combined_36 | 0.40639801 | 0.98775812 | 0.47279051 |
| combined_18 | 0.4274989 | 0.98775812 | 6.68E-19 |
| combined_30 | 0.43346934 | 0.98775812 | 5.88E-15 |
| combined_37 | 0.43572514 | 0.98775812 | 0.21518915 |
| combined_08 | 0.43809642 | 0.98775812 | 2.99E-17 |
| combined_34 | 0.43809642 | 0.98775812 | 0.45327438 |
| combined_28 | 0.44684108 | 0.98775812 | 0.30273402 |
| combined_00 | 0.47554013 | 0.98775812 | 0.44787677 |
| combined_06 | 0.49554741 | 0.98775812 | 9.97E-05 |
| combined_29 | 0.49554741 | 0.98775812 | 0.3039309 |
| combined_35 | 0.49554741 | 0.98775812 | 0.00057074 |
| combined_38 | 0.51742138 | 0.98775812 | 0.40715311 |
| combined_19 | 0.72772175 | 0.98775812 | 0.47279051 |
| combined_23 | 0.72772175 | 0.98775812 | 4.04E-18 |
| combined_27 | 0.72772175 | 0.98775812 | 1.38E-15 |

**Table S1. Module-trait relationships for combined network from astrocyte Ribotag data.** Significant association to EAE, region, or interaction was determined using factorial ANOVA. P-values for each term were adjusted for multiple comparisons using the Benjamini-Hochberg Procedure.


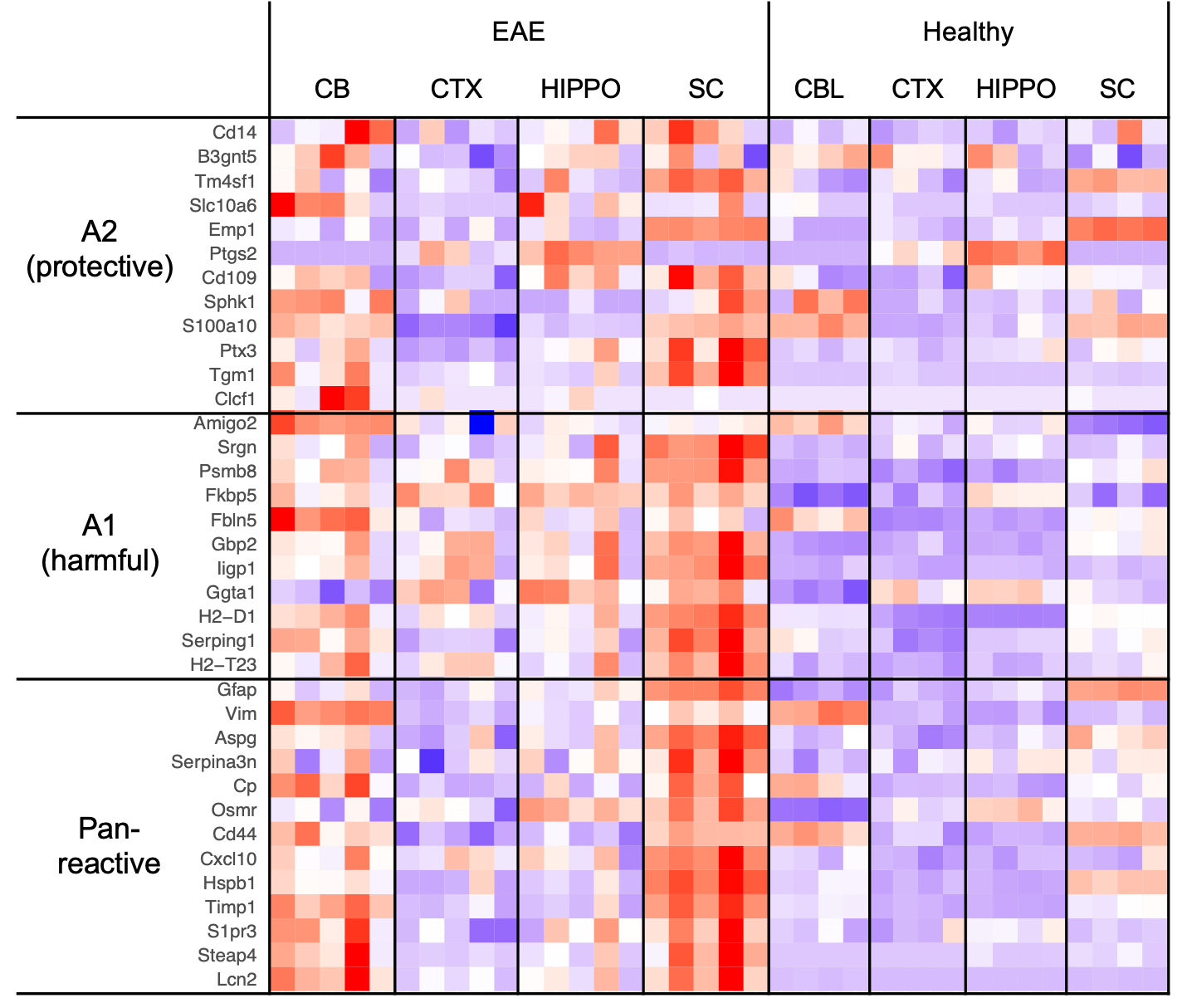


**Figure S3. Expression of reactive astrocyte-specific transcripts in astrocyte Ribotag data**. Heatmap showing the expression of reactive astrocyte transcripts from Liddelow et al. 2017 in the astrocyte Ribotag data. As expected, EAE astrocytes have evident upregulation of many reactive astrocyte transcripts, especially in the spinal cord and cerebellum.

**Figure S4. Head-to-head comparisons of 4 and 6 month versions of the turquoise module.** A) Alignment of modules 4m-4 and 6m-2. Both modules are sorted by descending intramodular connectivity (kWithin). Edges connect genes from 4m-4 to their location in 6m-2. Edges are colored based on their connectivity in 4m-4, which is largely preserved in 6m-2. B) Percent similarity (overlap/total) when the top N number of genes from each module are taken. Maximum percent similarity is 68% at 504 genes. C) Overlap at the max similarity from B. D) Correlation between intramodular connectivity (kWithin) of 4m-4 and 6m-2. E) Cumulative distribution function of connectivity for overlapping genes (black) and non-overlapping genes (green). The connectivity of overlapping genes is higher.

**Figure S5. Head-to-head comparisons of 6 and 8 month versions of the turquoise module.** A) Alignment of modules 6m-2 and 8m-1. Both modules are sorted by descending intramodular connectivity (kWithin). Edges connect genes from 6m-2 to their location in 8m-1. Edges are colored based on their connectivity in 6m-2, which is largely preserved in 8m-1. B) Percent similarity (overlap/total) when the top N number of genes from each module are taken. Maximum percent similarity is 65% at 618 genes. C) Overlap at the max similarity from B. D) Correlation between intramodular connectivity (kWithin) of 6m-2 and 8m-1. E) Cumulative distribution function of connectivity for overlapping genes (black) and non-overlapping genes (green). The connectivity of overlapping genes is higher.

**Figure S6. Head-to-head comparisons of 6 and 8 month versions of the turquoise module.** A) Alignment of modules 4m-4 and 8m-1. Both modules are sorted by descending intramodular connectivity (kWithin). Edges connect genes from 4m-4 to their location in 8m-1. Edges are colored based on their connectivity in 4m-4, which is largely preserved in 8m-1. B) Percent similarity (overlap/total) when the top N number of genes from each module are taken. Maximum percent similarity is 61% at 473 genes. C) Overlap at the max similarity from B. D) Correlation between intramodular connectivity (kWithin) of 4m-4 and 8m-1. E) Cumulative distribution function of connectivity for overlapping genes (black) and non-overlapping genes (green). The connectivity of overlapping genes is higher.
